# Biological properties of almond proteins produced by aqueous and enzyme-assisted aqueous extraction processes from almond cake

**DOI:** 10.1038/s41598-020-67682-3

**Published:** 2020-07-02

**Authors:** Thaiza S. P. de Souza, Fernanda F. G. Dias, Joana Paula S. Oliveira, Juliana M. L. N. de Moura Bell, Maria Gabriela B. Koblitz

**Affiliations:** 1 Department of Food Science and Technology, University of California, Davis, One Shields Avenue, Davis, CA 95616 USA; 2 Department of Food Science, Food and Nutrition Graduate Program, Federal University of the State of Rio de Janeiro, 296, Pasteur Avenue, Urca, Rio de Janeiro, RJ 29622290-240 Brazil; 3 Biological and Agricultural Engineering, University of California, Davis, One Shields Avenue, Davis, CA 95616 USA

**Keywords:** Biochemistry, Biotechnology, Computational biology and bioinformatics, Microbiology, Environmental sciences, Metabolic disorders

## Abstract

The almond cake is a protein-rich residue generated by the mechanical expression of the almond oil. The effects of the aqueous (AEP) and enzyme-assisted aqueous extraction processes (EAEP) on the biological properties of the almond cake protein were evaluated. Total phenolic content (TPC), antioxidant capacity, inhibitory effects against crucial enzymes related to metabolic syndrome, antimicrobial potential, and in vitro protein digestibility profile were assessed. EAEP provided the best results for antioxidant capacity by both ORAC (397.2 µmol TE per g) and ABTS (650.5 µmol TE per g) methods and also showed a high (~ 98%) potential for α-glucosidase inhibition. The AEP resulted in protein extracts with the highest lipase inhibition (~ 70%) in a dose-dependent way. Enzymatic kinetic analyses revealed that EAEP generated uncompetitive inhibitors against α-glucosidase, while EAEP, AEP, and HEX-AEP (used as control) generated the same kind of inhibitors against lipase. No protein extract was effective against any of the bacteria strains tested at antimicrobial assays. An in silico theoretical hydrolysis of amandin subunits corroborated with the results found for antioxidant capacity, enzyme inhibitory experiments, and antimicrobial activity. Digestibility results indicated that the digestive proteases used were efficient in hydrolyzing almond proteins, regardless of the extraction applied and that HEX-AEP presented the highest digestibility (85%). In summary, EAEP and AEP skim proteins have the potential to be used as a nutraceutical ingredient. The biological properties observed in these extracts could help mitigate the development of metabolic syndrome where EAEP and AEP skim proteins could be potentially used as a prophylactic therapy for diabetes and obesity, respectively.

## Introduction

The consumption of almonds (*Prunus dulcis*, syn. *Prunus amygdalus*) has been associated with various health benefits. Its antioxidant activity is usually attributed to the presence of α-tocopherols and polyphenols, with the latter being associated with reduced risk of metabolic syndrome—through the regulation of postprandial hyperglycemia and reducing the occurrence of diabetes *mellitus* type II—and potential antimicrobial effect^1^. Almond polyphenols are mainly found at the lipid interface and contribute to improving whole almonds shelf-life due to their antioxidant and antimicrobial activities^2^. In addition, almonds are considered a good source of protein, although methionine, lysine, and threonine are essential limiting amino acids. Almond's major storage protein is called amandin and represents about 70% of the total soluble proteins in the seed. Amandin is formed by two subunits: prunin-1 and prunin-2, which are composed of basic (20–22 kDa) and acidic (42–46 kDa) polypeptides^3^.

Because almonds are a good source of lipids and proteins, prior removal of oil is needed to produce defatted protein fractions. The mechanical expression of almond oil generates a protein-rich cake which is commonly used as animal feed. While the composition of the cake is intrinsically related to the pressing conditions, which in turn dictates the extraction efficiency, oil and protein contents of 16 and 37% have been observed for the almond cake, respectively^4^. The residual oil in the protein-rich cake is commonly extracted by the use of hexane, a practice that has raised environmental, safety (flammability), and health (neurotoxic effects) concerns^5^. These concerns, along with increasingly restrictive regulations, have prompted the search for more environmentally friendly extraction approaches^6^. Aqueous (AEP) and enzyme-assisted aqueous extraction processes (EAEP) are environmentally friendly strategies that can replace the use of hexane and enable the simultaneous extraction of oil, protein, and carbohydrate from many oil-bearing materials^7^. In addition, the EAEP offers the possibility of generating fractions with improved functionality and biological properties^8^. Research has shown that protein hydrolysis may generate bioactive peptide, improving the functional and biological properties of the original proteins^9^. Bioactive peptides have been associated with various biological properties such as antioxidant, antihypertensive, antimicrobial, antithrombotic, hypocholesterolemic, and immunomodulatory functions^10^. Valorization of the almond cake, as many other food byproducts, could be achieved by the tailored extraction of its major constituents (oil, proteins, and bioactive compounds) for subsequent use in food, feed, fuel, and nutraceutical applications. These compounds may be used in the formulation of functional foods or to improve the nutritional characteristics of other food products while contributing to the reduction of food waste^11^. The search for bioactive compounds (i.e., bioactive peptides) in agricultural and industrial byproducts has been increasing, as they bear substances with properties of interest to the food and pharmaceutical industries^12^.

The overall goal of this study was to evaluate the effects of the aqueous (AEP) and enzyme-assisted aqueous extraction processes (EAEP) on the biological properties of the skim fraction (protein-rich fraction) produced from the almond cake. The specific objectives of this study were to evaluate the in vitro protein digestibility, the total phenolic content, and the bioactive properties (antioxidant capacity, inhibitory effect against α-glucosidase, and pancreatic lipase, and antimicrobial activity) of the skim fractions generated from the AEP and EAEP of almond cake. These properties were compared with the skim fraction produced by the AEP of the hexane-defatted almond cake (used as a control). Therefore, this work aimed to evaluate the effects of two environmentally friendly extraction processes (AEP and EAEP) to generate proteins with improved biological functions from an underutilized food byproduct, ultimately leading to potential health benefits.

## Materials and methods

### Materials

Cold pressed almond cake was supplied by Blue Diamond (Sacramento, CA, USA). For EAEP, an endoprotease (FoodPro Alkaline Protease from *Bacillus licheniformis*; Danisco—NY, USA) was used. Hexane, Folin-Ciocalteu reagent, gallic acid, 2,20–Azino-bis (3-ethylbenzothiazoline-6-sulphonic acid) (ABTS), potassium persulfate, Trolox, fluorescein, 2,2′-Azobis (2 methylptopionamidine) dihydrochloride (AAPH), α-glucosidase, p-nitrophenyl-α-D-glucopyranoside (p-NPG), lipase from porcine pancreas type II (EC 3.1.3), 4-nitrophenyl laurate (p-NP-Laurate) were purchased from Sigma-Aldrich (St. Louis, USA). The pathogenic strains tested were supplied by Fiocruz (Rio de Janeiro, Brazil).

### Aqueous (AEP) and enzyme-assisted aqueous extraction processes (EAEP)

Optimum extraction conditions identified by Souza et al.^4^ were scaled up for the AEP (aqueous extraction process), EAEP (enzyme-assisted aqueous extraction process), and HEX-AEP (solvent aqueous extraction process). Briefly, the almond cake (oil 16.25 g.100 g^−1^, protein 37.20 g.100 g^−1^, and moisture 9.04 g.100 g^−1^) was dispersed in distilled water in a ratio of 1:12.8 (w/v) into a 10-L jacketed glass reactor (Chemglass, NJ, USA). For HEX-AEP, the almond cake was previously defatted with hexane in a Soxhlet device for 6 h at 68 °C. Process conditions were: 50 °C, pH 9.0, 120 rpm, reaction time—2 h (AEP and HEX-AEP), or 1 h and addition of alkaline protease 0.85 g.100 g^−1^ (EAEP). The slurry was centrifuged at 3,000 × *g* for 30 min at 25 °C and the supernatant was allowed to cool down overnight at 4 °C to separate skim from cream fraction when the latter was formed. Approximately 16 kg of each skim fraction was freeze-dried (Lyophilizer—Virtis, CA, EUA) generating about 650 g of freeze-dried sample, as each extraction was done in duplicate.

### Total phenolic content (TPC)

Samples of 1 g of the freeze-dried skim proteins were mixed with 10 mL of distilled water, ethanol (100%), or methanol (100%), stirred for 1 h and centrifuged (5,000 × g/ 5 min)^11^. TPC was quantified by the Folin-Ciocalteu reagent method as modified by Singleton et al.^13^. Readings were performed in a microplate reader (FlexStation 3; Molecular Devices, CA, USA) and the absorbance was recorded at 750 nm. TPC was calculated from the equation of a standard curve of gallic acid ranging from 5 to 130 µg. mL^−1^ (R^2^ = 0.9982, y = 0.0102x—0.0215). TPC was expressed as mg of gallic acid equivalents (GAE) per 100 g of dry sample.

### Antioxidant capacity

#### ABTS assay

ABTS assay was based on Ngoh and Gan^10^ with slight modifications. Samples (10 µL), at concentrations ranging from 0.5 to 5 mg mL^−1^ (in distilled water) were mixed with 190 µL of diluted ABTS reagent. Absorbance was recorded at 720 nm, in a microplate reader, at 37 °C, in triplicate. Antioxidant activity was calculated from the equation of a standard curve of Trolox ranging from 50 to 750 µM (R^2^ = 0.979, y = -0.0004x—0.0117). The antiradical activity was expressed as µMol of Trolox equivalent per g of sample.

#### ORAC assay

The ORAC method was performed according to Zulueta et al.^14^. Samples (80 µL) at concentrations ranging from 0.025 to 0.200 mg mL^−1^ (in distilled water), PBS (blank) or Trolox (standard) were mixed with 80 µL of fluorescein (78 nM) followed by 40 µL of APPH (221 mM). The fluorescence was measured every minute for 90 min (excitation—485 nm; emission—535 nm). A calibration curve using Trolox solutions (10–120 µM) (R^2^ = 0.9891, y = 62.444x + 734.12) was used and the antiradical activity was expressed as µMol of Trolox equivalent (TE) per g of sample using Eq. 1.

1 $${\text{ORAC}}~\left( {\mu {\text{M}}~{\text{TE g}}^{{ - 1}} } \right) = ~\left. {\left( {~\frac{{~{\raise0.7ex\hbox{${\left( {AUCs - AUCb} \right) - b}$} \!\mathord{\left/ {\vphantom {{\left( {AUCs - AUCb} \right) - b} a}}\right.\kern-\nulldelimiterspace} \!\lower0.7ex\hbox{$a$}}}}{C}} \right.} \right),$$

where AUCs and AUCb are the areas under the curves of the sample and blank, respectively; a is the intercept and b is the slope from the standard curve, and C is the sample concentration tested in the experiment.

### Enzymatic inhibition

#### α-Glucosidase inhibition assay

The α-glucosidase inhibitory activity was evaluated according to Ibrahim et al.^15^, with slight modifications. Samples diluted in water (50 μL) at final concentrations of 0.5 to 40 mg mL^−1^ or ultrapure water (control) were incubated with 25 μL of 0.5 U.mL^−1^ α-glucosidase solution in PBS (100 mM, pH 6.8), at 37 °C, for 1 h. After pre-incubation, 25 μL of p-NPG substrate solution (5 mM) in PBS (100 mM, pH 6.8) was added, and the mixture was incubated at 37 °C, for 30 min. One hundred microliters of glycine–NaOH buffer (pH 10.0) were added to stop the reaction. The absorbance was measured at 405 nm in a microplate reader. The inhibitory activity was expressed as a percentage of a control sample (without the inhibitors). The α-glucosidase inhibitory activity was calculated by using Eq. 2.

2 $$\alpha - {\text{glucosidase~}}\;{\text{inhibitory~}}\;{\text{activity}}~\left( \% \right) = ~\left. {\left( {1 - ~\frac{{As}}{{Ac}}} \right.} \right)~ \times ~100~,$$

where As and Ac are absorbances of sample and control, respectively.

#### Pancreatic lipase inhibition assay

The pancreatic lipase inhibitory activity was evaluated based on the method described by McDougall et al.^16^, adapted to a 96-well microplate. Samples (15 μL) at final concentrations of 5 to 10 mg mL^−1^ or ultrapure water (control) were incubated with 60 μL of pancreatic lipase (10 mg mL^−1^) solution in Tris–HCl buffer (100 Mm, pH 8.2), at 37 °C, for 30 min. After pre-incubation, 135 μL of p-NP-laurate (2.5 mM, in 5 mM sodium acetate buffer, pH 5.0, with 1% Triton X-100) was added to start the reaction and was incubated at 37 °C, for 2 h. The absorbance was measured at 405 nm using a microplate reader. The inhibitory activity was expressed as a percentage of a control sample (without the inhibitors). The pancreatic lipase inhibitory activity was calculated by using Eq. 3.

3 $${\text{pancreatic lipase inhibitory activity }}\left( {\text{\% }} \right) = \left. {\left( {1 - \frac{As}{{Ac}}} \right.} \right) \times 100 ,$$

where As and Ac are absorbances of sample and control, respectively.

#### Kinetics of enzyme inhibition

To identify the type of inhibition exerted by the protein extracts on α-glucosidase and pancreatic lipase, an enzyme inhibition kinetic experiment was performed according to Ibrahim et al.^15^. For the α-glucosidase and pancreatic lipase inhibition assays, a range of concentrations from 0.15 to 5.0 mM of p-NPG and 0.05 to 2.5 mM of p-NP-Laurate was used. Lineweaver–Burk plots were used to determine the kinetic constants, K_m_ (Michaelis constant) and V_max_ (maximum velocity).

#### In silico theoretical hydrolysis

In silico digestion of prunin-1 and prunin-2 sequences by the subtilisin enzyme (EC 3.4.21.62) was conducted using the enzymatic action tool incorporated into the BIOPEP platform^17^. Prunin-1 and prunin-2 sequences were taken from the UniProt platform (accession number Q43607 and E3SH29, respectively). The antioxidant peptides were evaluated by the BIOPEP biological activity database limiting the activities evaluated to antioxidants. For α-glucosidase and pancreatic lipase inhibitory peptide profiles, the criteria suggested by Ibrahim et al.^18^ and Ngoh and Gan^10^ was used.

#### Antimicrobial assay

For antimicrobial assays, the gram-positive bacterial strains *Staphylococcus aureus* (NCQS 00,402), *Bacillus cereus* (NCQS 00,445) and *Listeria monocytogenes* (NCQS 00,673) and the gram-negative bacteria strains *Escherichia coli* (NCQS 00,595) and *Salmonella enterica* subsp. *enterica* (NCQS 00,236). The bacterial strains were consecutively sub-cultured with 24 h intervals. After the activation, were made a bacterial suspension, which was adjusted to 0.5 McFarland scale.

#### Agar disk diffusion method

The antimicrobial effects were firstly determined by the agar disk diffusion method according to Balouiri et al.^19^ and Kim et al.^20^, with modifications. Filter paper discs were placed on the tryptic soy agar surface. Ten μL of the diluted sample in water (20 mg mL^−1^), the standard (amoxicillin, 2 mg mL^−1^) or the blank (saline solution) were added on top of discs. Petri dishes were incubated for 24 h and observed. A clear zone (halo) characterizes a positive result and no halo formed means negative result for bacterial growth inhibition.

#### Broth dilution method

The broth microdilution test according to Balouiri et al.^19^ and Kim et al.^20^ was performed with modifications. Serial two-fold micro dilutions were prepared in a 96-well microplate. Fifty μL of the sample (diluted in water) or the standard (amoxicillin) and the same volume of overnight bacterial suspension stains, in the range of 0.5 to 70 mg mL^−1^ (final concentration in well) were added in each well. The microplates were incubated at 37 °C, for 24 h. Bacterial growth was estimated by absorbance readings at 660 nm.

### Digestibility

#### In vitro protein digestibility

Protein digestibility was measured as described by Roman et al.^21^ and Bornhorst and Singh^22^. The composition of the digestive solutions is presented in Table 1. Five grams of liquid skim fractions were mixed with 3.33 mL of SSF (Simulated Saliva Fluid) and vortexed. Subsequently, 6.66 mL of SGF (Simulated Gastric Fluid) was added. Afterward, the pH was adjusted to 3.0 and the samples were placed into a water-bath (37 °C, 140 rpm, 2 h). Then, 10 mL of SIF (Simulated Intestinal Fluid) was added, and the pH was adjusted to 7.0. The samples were incubated into a water-bath at 37 °C, 140 rpm, for 2 h. To stop the digestion, samples were heated in a water bath at 85 °C for 3 min. TCA (12 g.100 g^−1^) was added in a 1:1 (v/v) proportion and the samples were centrifuged at 4,000 rpm for 30 min at 4 °C. Total nitrogen (NT) and nonprotein nitrogen (NPN)—soluble fraction after TCA (12 g.100 g^−1^) precipitation—were measured in the samples, by the Dumas method using a conversion factor of 5.18 (Vario MAX cube, HE, DE) before and after the digestion. In vitro protein digestibility was calculated by using Eq. 4^23^.

**Table 1:** Composition of the simulated fluids.

Simulated Saliva Fluid (SSF)	Final concentration (mg mL^−1^)	Final pH
Mucin	1.0	7.0
NaCl	0.117	
KCl	0.149	
NaHCO_3_	2.1	

Simulated Gastric Fluid (SGF)	Final concentration (mg mL^−1^)	Final pH
Pepsin	0.75 (2000 U/mL)	1.8–2.0
Gastric mucin	1.5	
NaCl	8.78	

Simulated Intestinal Fluid (SIF)	Final concentration (mg mL^−1^)	Final pH
Pancreatin	8.0 (800 U/mL)	7.0
Bile extract	10.0	
NaHCO_3_	16.8	

4 $$D \left(\%\right) =\frac{{NPN}_{after }- {NPN}_{before }- { NPN}_{enzymes (blank) }}{{NT}_{before }-{ NPN}_{before }} x 100$$

where NPN_after_ = protein after digestion, NPN_before_ = protein before digestion, NPN_enzyme_ = enzyme blank and NT_before_ = total protein before digestion.

#### In vitro digestibility evaluation by SDS-page

SDS-PAGE was used to evaluate different stages during the digestion of each skim. The protein profile was assessed as described by Laemmli^24^ with few modifications. Thirty micrograms of protein were loaded onto the precast 12% acrylamide Criterion TGX Precast gel. A low range SDS-PAGE standard (14.4–97.4 kDa) was used as a molecular mass marker. The gel was imaged using a Gel Doc EZ Imager system and Image Lab software (Bio-Rad Laboratories, CA, USA).

### Statistical analyses

The experiments were performed at least in triplicate, and the results were expressed as mean ± standard deviation (SD) of the replicates. Analyses of variance (ANOVA) were performed, followed by the Tukey test using Graph Pad Prism 5.0 (version 5.04, GraphPad Software, CA, USA). Associations between antioxidant capacity and phenolic compounds were assessed by Pearson correlation. Significant differences were established at p < 0.05.

## Results and discussion

### Total phenolic content (TPC)

**Figure 1:**
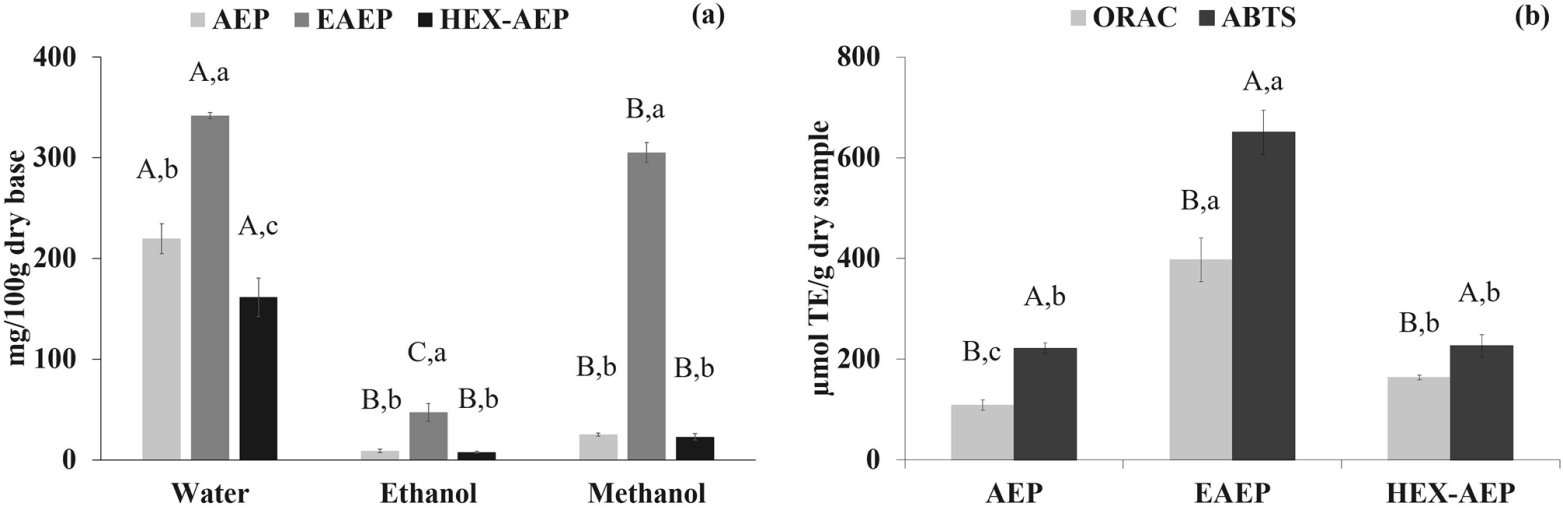
(**a**) Total phenolic content extracted by water, ethanol, and methanol. Different capital letters indicate significant difference (p < 0.05) among the solvents (water, ethanol, and methanol) for the same skim and different lowercase letters indicate significant difference (p < 0.05) between the different skims (AEP, EAEP, and HEX-AEP) within the same solvent; (**b**) Antioxidant capacity evaluated by the ORAC and by the ABTS methods. Different capital letters indicate a significant difference (p < 0.05) between the antioxidant activity analyses (ORAC and ABTS) for the same skim and different lowercase letters indicate a significant difference (p < 0.05) between the different skims for the same antioxidant activity evaluation method. Data represent the mean ± one standard deviation of triplicates.

The effects of different extraction conditions on the content of phenolic compounds in the skims are shown in Fig. 1a. It can be observed that the EAEP generated a skim fraction with the highest TPC, regardless of the solvent used to extract the phenolics. Among the solvents evaluated, higher phenolic extraction was achieved when using water as a solvent. TPC in the skims ranged from 7.7 to 342 mg GAE/100 g dry basis, depending on the solvent used and the type of extraction used. Using water as a solvent, TPC of 342; 219.7; and 161.4 mg GAE per 100 g dry sample were observed for the EAEP, AEP, and HEX-AEP skim fractions, respectively. In the present study, the cold pressed cake used was produced from whole natural almonds. When comparing with the literature, our results are in agreement with the values obtained by Milbury et al.^25^, who observed concentrations from 126.8 to 240.8 mg GAE per 100 g, depending on the almond variety; and with Bolling^26^, that reported 190 mg GAE per 100 g, both studies on the whole almond.

The solvent polarity plays an important role in the recovery of polyphenols from different matrices, as it affects the solubility of the phenolic compounds^27^ and, according to Abarca-Vargas et al.^28^, water, ethanol, methanol, and their mixtures are the most frequently solvents used for TPC extraction. In this study, TPC yield related to the solvent as follows: water > methanol > ethanol. The results suggested that the higher the polarity of the solvent, the higher the extractability (Polarity Index: water = 9.0; methanol = 6.6 and ethanol = 5.2)^28^. A similar trend was also found by Gomaa^29^, where higher TPC yields in bitter almonds and sweet apricots were achieved when using water as a solvent, compared to methanolic and ethanolic extracts. In a previous review regarding almond polyphenols, Bolling et al.^26^ showed that the most abundant polyphenols were proanthocyanidins (epicatechin and catechin), hydrolyzable tannins (gallotannins, ellagitannins, and phlorotannins), and flavonoids (anthocyanidins, flavan-3-ols, flavonols, flavanones, and biflavone). Proanthocyanidins have also been identified as primary phenolic compounds in whole almonds by other authors^30^. Zam et al.^31^ evaluated TPC at pomegranate’s peel and observed that water archived the highest extraction yield of polyphenols in general and of proanthocyanins in particular. The high extractability when using water as solvent can be related to the weakening of the hydrogen bonds in aqueous solutions^32^. This observation may explain the higher solubility of the phenolic compounds from the almond cake skim fraction in water than in the other solvents.

In addition to the solvent used, the use of enzyme or not during the extraction also influenced the total recovery of phenolic compounds from the skims. The EAEP skim showed the highest yields of TPC compared with the AEP, which is likely due to the effect of the protease action during the extraction. Phenolic compounds can bind to proteins and carbohydrates, therefore hydrolysis of these complexes by proteases might help the release of entrapped phenolics^33^. Pinelo et al.^33^ studied the release of phenolic compounds from apple skin and observed that proteases favored their release. In the present study, the phenolic compounds that were inaccessible to the AEP and HEX-AEP skims were possibly released after the addition of the protease in the EAEP, enabling their solubilization into the solvent. Among the sample, HEX-AEP presented the lowest phenolic content in water. This might be related to the previous fat removal of HEX-AEP, where the sample was exposed to a mild heat treatment at ~ 68 °C and extraction temperature above 65 °C may lead to phenolics degradation^34^.

### Antioxidant activity

The antioxidant capacity was assessed in the extracted proteins using the ABTS and ORAC assays (Fig. 1b). EAEP skim showed the highest antioxidant capacity for both ORAC (397.2 µmol TE per g) and ABTS (650.5 µmol TE per g) methods. HEX-AEP (163.5 µmol TE per g) showed higher antioxidant capacity followed by AEP (108.7 µmol TE per g) by the ORAC method, while HEX-AEP (226.7 µmol TE per g) and AEP (221.7 µmol TE per g) showed no significant difference by the ABTS method. Although both methods followed the same trend and presented an extremely high (R^2^ = 0.98) correlation coefficient by Pearson’s evaluation, in general, the values found by the ABTS assay were higher than by the ORAC method. This variation is probably related to the differences in the antioxidant capacities measured by the two methods. ABTS or TEAC (Trolox equivalent antioxidant capacity) is a method based on the measurement of the electron transfer capacity of the antioxidant evaluated whereas ORAC (oxygen radical antioxidant capacity) is an assay that is based on the quantification of the hydrogen atom transfer capacity of the antioxidant evaluated^14^. Thus, the same antioxidant mixture may have a high capacity to transfer electrons and a low capacity to transfer hydrogen atoms or vice versa, resulting in significantly different values for the two methods tested. EAEP showed 3.6 and 2.4 times more antioxidant capacity than AEP and HEX-AEP skins respectively when the ORAC method was applied, whereas this feature was 2.9 times higher than AEP and HEX-AEP when the ABTS assay is considered.

Higher antioxidant capacity of EAEP skim can be in part explained by the highest TPC in this sample (Fig. 1a), as proanthocyanidins, already identified in whole almonds, are recognized for increasing antioxidant capacity^26,30^ and, according to Pearson’s correlation coefficient, ABTS (R^2^ = 0.94) and ORAC (R^2^ = 0.87) assays strongly correlated with the TPC for each sample. Thus, another possible explanation for the higher activity in EAEP skim may be the formation of potential antioxidant peptides during the enzymatic extraction (Fig. 4). The protease used for EAEP processing might have hydrolyzed the proteins present in the almond cake and later in the skim fraction, generating peptides with antioxidant activities. In fact, the theoretical hydrolysis (Supplementary Table S1) of the most important storage protein of almonds (amandin) revealed the formation of 4 different antioxidant peptides (HL, IY, VY, PHW). Some studies have identified antioxidant peptides from several plant-based proteins, such as peanuts^8^ and okara^35^. These studies correlated the antioxidant capacity with the degree of hydrolysis (DH %), whereas the higher DH %, the higher the antioxidant capacity. The DH % of the skims were evaluated in a previous study from our group and 23, 1.8, and 1.3% were achieved for the EAEP, AEP, and HEX-AEP skim fractions. The higher DH % of the EAEP skim may have contributed to the higher antioxidant capacity observed, which is in agreement with the studies above cited. AEP and HEX-AEP skims showed no significant differences for DH %, nor for TCP, when methanol and ethanol were applied, or for the antioxidant capacity measured by the ABTS assay.

To the best of our knowledge, this is the first study to investigate the effects of extraction conditions on the antioxidant properties of almond cake protein extracts. Interestingly, our results showed the use of an enzyme in the EAEP can improve the antioxidant capacity of the extracted protein, as revealed by the ABTS and ORAC assay results.

### α-glucosidase and pancreatic lipase inhibition

**Figure 2:**
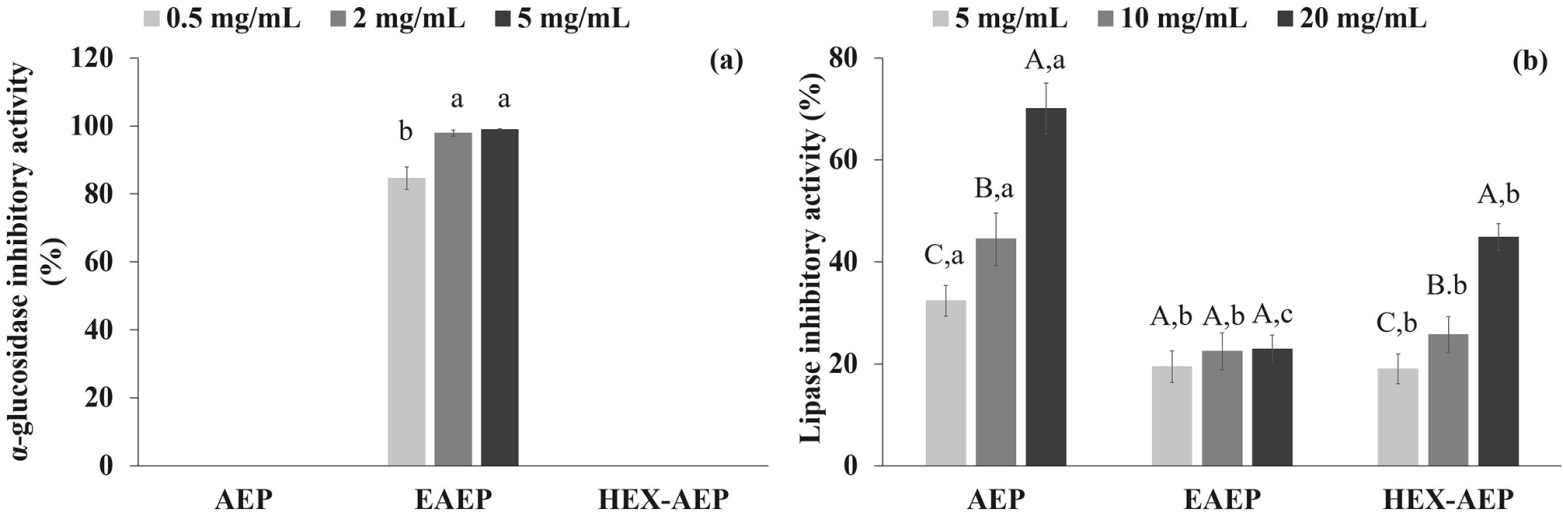
(**a**) α-glucosidase inhibitory activity of EAEP skim. Different letters indicate a significant difference (p < 0.05) among the concentrations evaluated. (**b**) Lipase inhibitory activity of AEP, EAEP, and HEX-AEP skims. Different capital letters indicate a significant difference (p < 0.05) among the concentrations evaluated (5, 10 and 20 mg mL^−1^) for the same skim and different lowercase letters indicate a significant difference (p < 0.05) among AEP, EAEP, and HEX-AEP skims for the same concentration. Data represent the mean ± one standard deviation of triplicates.

**Figure 3:**
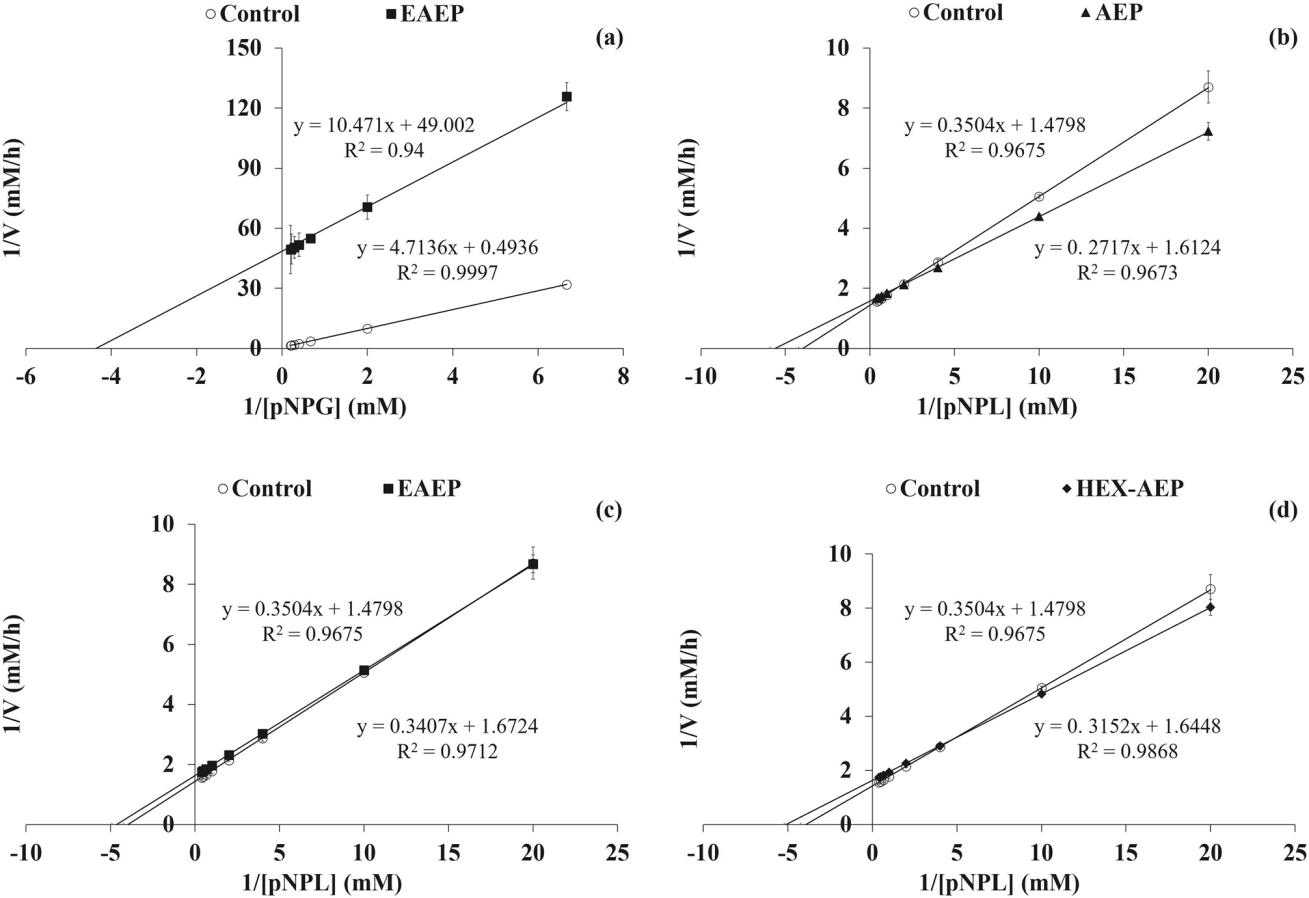
(**a**) Lineweaver–Burk plots of α-glucosidase inhibition for control (no inhibitor) and EAEP skim (2 mg mL^−1^); (**b**) Lineweaver–Burk plots of lipase inhibition for control (no inhibitor) and AEP (20 mg mL^−1^); (**c**) EAEP (5 mg mL^−1^) and (**d**) HEX-AEP (20 mg mL^−1^) skims.

A therapeutic approach to prevent diabetes and obesity is to inhibit digestive enzymes such as α-glucosidase and pancreatic lipase. Inhibitors targeting these enzymes prevent the uptake of glucose from complex dietary carbohydrates and free fatty acids from triacylglycerols to be absorbed into the body^9,15^. Preliminary tests evaluated potential inhibitory effects of the extracted proteins against α-glucosidase (Fig. 2a) or pancreatic lipase (Fig. 2b) as well as the lowest concentration that exerted the highest inhibition. It seems evident that, among the samples tested, only the EAEP skim presented α-glucosidase inhibitory activity (Fig. 2a). In addition to the concentrations evaluated in Fig. 2a, higher concentrations of AEP and HEX-AEP skims (10, 20, and 40 mg mL^−1^) were also evaluated but showed no activity. The EAEP skim, on the other hand, showed high inhibition, even in the lowest concentration tested: 0.5 mg mL^−1^ inhibited 84.6% of the α-glucosidase activity while 2.0 mg mL^−1^ inhibited 97.8% of the α-glucosidase activity, with no significant improvement for higher concentrations (5.0 mg mL^−1^—98.9%). Unlike α-glucosidase, the pancreatic lipase was inhibited by all the samples tested (Fig. 2b). AEP skim showed the highest dose-dependent inhibitory activity followed by HEX-AEP skim, which showed little over half of AEP’s inhibitory activity. EAEP skims showed the lowest inhibition of the pancreatic lipase, which remained unchanged (p > 0.05) regardless of the concentration tested. A kinetic study was performed to verify the type of inhibition taking place in the skim fraction, applying the least concentration of each sample that provided the highest inhibition (Fig. 3).

The enzymes tested in this study—α-glucosidase and pancreatic lipase—are a part of the digestion of carbohydrates and triacylglycerols in the digestive tract^35^. Alpha-glucosidase is involved in the digestion of starch, catalyzing the hydrolysis of oligo- and disaccharides to release free molecules of glucose^9,15^. Therefore inhibitors of α-glucosidase aid to control postprandial hyperglycemia by modulating the digestion rate of complex carbohydrates such as starch and may be employed in the prevention of diabetes and other associated diseases^15^.

In this study, 2 mg mL^−1^ of EAEP skim inhibited ~ 98% of the α-glucosidase activity, while AEP and HEX-AEP skims did not present an inhibitory effect. These results may be explained by the generation of bioactive peptides, through the alkaline protease activity in the enzymatic extraction (EAEP) compared with the aqueous extraction process (AEP nor HEX-AEP) where no enzyme was used during the extraction. Bioactive peptides are encrypted within the primary structure of proteins and may be released by proteolysis. These peptides may act as inhibitors of metabolic enzymes and present potential use as therapeutic agents against specific diseases. Ibrahim et al.^18^ identified the structural and physicochemical requirements to design an active α-glucosidase inhibitory peptide. According to their findings, α-glucosidase inhibitory activity is related to short peptides, with 3 to 6 amino acid residues, containing a hydroxyl or basic side-chain amino acid at the N-terminus and a proline residue closer to the C-terminus with methionine or alanine occupying the final C-terminal position. The theoretical hydrolysis of amandin subunits (Supplementary Table S2) showed the formation of 13 different peptides with at least one of the above characteristics. It is therefore possible that, after proteolysis, some bioactive peptides were released from the almond cake proteins, leading to the high inhibitory activity of EAEP skim against α-glucosidase. Awosika and Aluko^9^ reported that 20 mg mL^−1^ of yellow field pea peptides inhibited up to 53.3% α-glucosidase activity and Oseguera-Toledo et al.^37^ evaluated peptide fractions from pinto Durango beans and observed inhibitory activity of 76.4% against α-glucosidase. This study is, however, the first report of such activity for almond protein hydrolysates from the almond cake.

Pancreatic lipase is responsible for the digestion and absorption of dietary fat through the hydrolysis of the triacylglycerols to glycerol and fatty acids^9,36^. The inhibition of pancreatic lipase prevents the breakdown of dietary fat into fatty acids, therefore reducing their absorption in the gut, which may be a viable approach to the control of hyperlipidemia and obesity^36^. Some in vitro studies reported plants with a high concentration of fat or high content of tannins as pancreatic lipase inhibitors^16,36^. Proanthocyanidins, already reported in almonds^30^, are among the primary active tannins and their activity was attributed to their ability to bind proteins, leading to the complexation and precipitation of the enzymes^38^. The same authors, however, claim that the mechanism of polyphenolic compounds on pancreatic lipase inhibition remains unclear. In the present study, all skim fractions showed some degree of lipase inhibition: AEP skim exhibited the highest inhibitory percentage, followed by HEX-AEP and EAEP skims. The TPC in this same samples did not follow this trend; on the contrary, EAEP skim showed the highest TPC of all samples (Fig. 1a).

**Figure 4:**
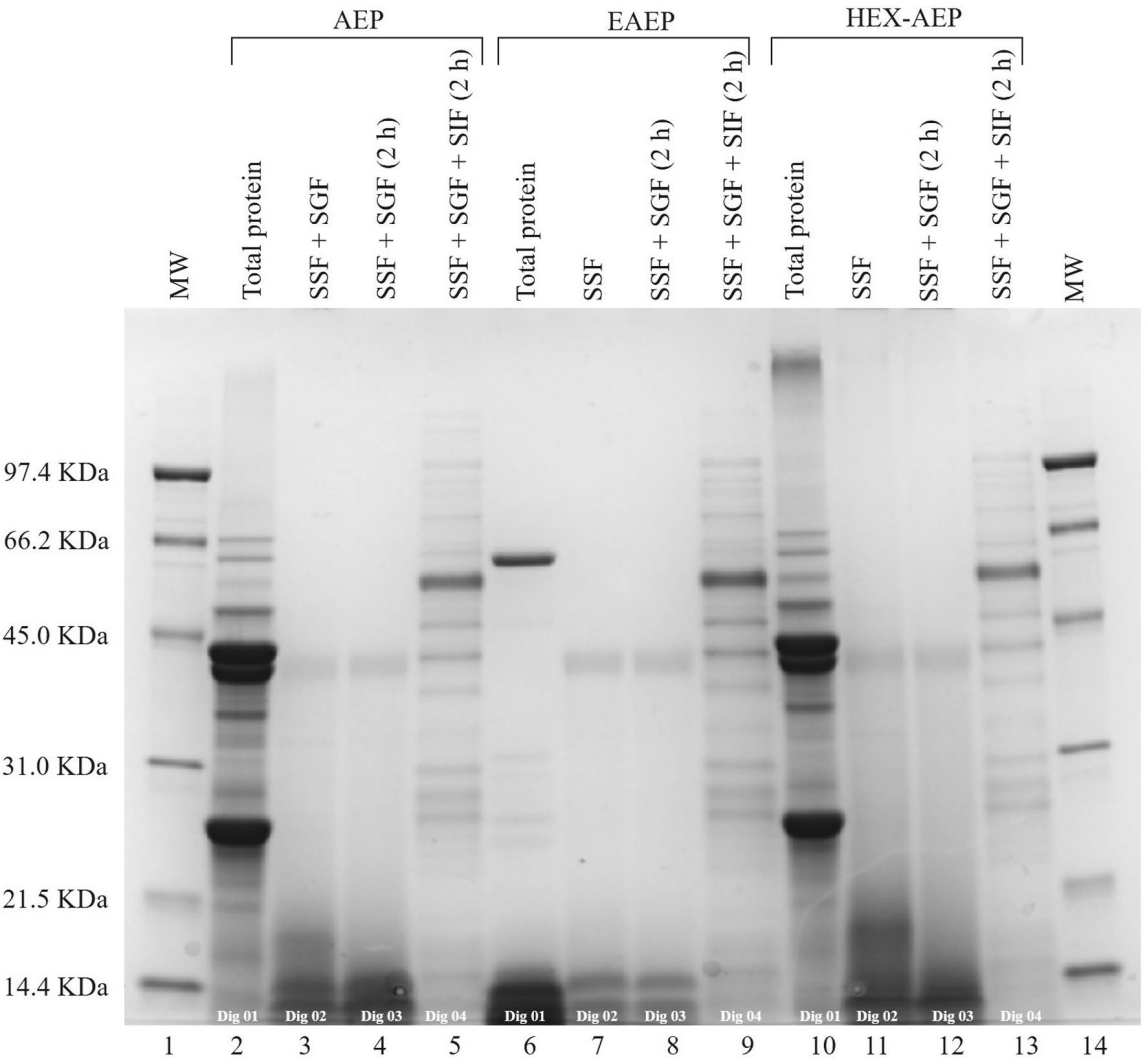
SDS-PAGE (12% Criterion TGX Precast Gels) analysis of the different stages of in vitro digestion of EAEP, AEP, and HEX-AEP. Samples derive from the same experiment and the gel was processed in parallel. The gel was imaged with a Gel Doc EZ (Bio-Rad, USA) using the Image Lab software. lane (1) Molecular mass marker standards (14.4–97.4 kDa); lane (2) AEP total protein; lane (3) AEP plus SSF and SGF; lane (4) AEP plus SSF, and SGF after 2 h reaction; lane (5) AEP plus SSF, SGF, and SIF after 2 h reaction; lane (6) EAEP total protein; lane (7) EAEP plus SSF and SGF; lane (8) EAEP plus SSF, and SGF after 2 h reaction; lane (9) EAEP plus SSF, SGF, and SIF after 2 h reaction; lane (10) HEX-AEP total protein; lane (11) HEX-AEP plus SSF and SGF; lane (12) HEX-AEP plus SSF, and SGF after 2 h reaction; lane (13) HEX-AEP plus SSF, SGF, and SIF after 2 h reaction; lane (14) MW marker standards (14.4–97.4 kDa).

Information about lipase inhibition by proteins or peptides is scarce in the literature, and there is even less information on pancreatic lipase activity inhibition by food protein-derived peptides^9^. However, a few recent studies reported peptides as pancreatic lipase inhibitors: Ngoh and Gan^10^ found bioactive peptides in pinto beans that inhibited the lipase activity in a range between 23 to 87% and Stefanucc et al.^39^, who discovered novel tripeptides as lipase inhibitors, observed inhibitions from 50 to 100 mg Orlistat (i.e., standard drug to treat obesity) equivalent per g of sample, depending on the peptide sequences. Ngoh and Gan^10^ verified, through docking analysis, that most of the amino acid residues of the peptides involved in pancreatic lipase inhibition were hydrophobic amino acids such as proline (P), leucine (L), glycine (G), phenylalanine (F), alanine (A) and methionine (M). The theoretical hydrolysis of amandin (Supplementary Table S3) showed the formation of 17 highly hydrophobic peptides, due to the presence of these amino acid residues. Awosika and Aluko^9^ evaluated yellow field pea protein hydrolysates and observed that trypsin and alcalase generated peptides with higher lipase inhibition capacity than chymotrypsin and pepsin. Alcalase is a subtilisin similar to the alkaline protease used in the EAEP. Although the EAEP skim showed the highest TPC and peptide content, this sample was not the most efficient for lipase inhibition. This inefficiency may have occurred due to the high degree of hydrolysis (DH = 23%) of the EAEP skim, which possibly generated peptides with a different profile from those naturally present in the AEP and HEX-AEP skims (Fig. 4). These results indicate that the AEP skim is a good candidate for use as a pancreatic lipase inhibitor, while the EAEP skim could be used as a highly efficient α-glucosidase inhibitor.

### Kinetics of enzyme inhibition

Kinetics experiments were performed to reveal the mechanisms of action of the different skim samples on α-glucosidase and pancreatic lipase inhibition, applying the least concentration of each sample that provided the highest inhibition (Fig. 3): for α-glucosidase, 2 mg mL^−1^ of the EAEP skim was applied; for lipase 20 mg mL^−1^ of the AEP or of HEX-AEP skims and 5 mg mL^−1^ of EAEP skim were tested. Lineweaver–Burk plots revealed that EAEP skim behaved as an uncompetitive inhibitor for both, α-glucosidase and pancreatic lipase (Table 2). Likewise, AEP and HEX-AEP skims also behaved as uncompetitive inhibitors of pancreatic lipase (Table 2). The results showed that the lines (control vs. inhibitors) intersected at both y-axis and x-axis, at different points, in the Lineweaver–Burk plots (Fig. 3). This indicates that, in the presence of the inhibitor, both the Michaelis constant (Km) and the maximum velocity (Vmax) decreased when compared to the control. These results were confirmed by calculating the apparent Km and Vmax (Table 2). Inhibition of pancreatic lipase by AEP and HEX-AEP, however, showed inhibition graphs that cross before reaching the y-axis. This kind of behavior was related by Park et al.^40^ to competitive inhibition, although without the corroboration of Km or Vmax values.

**Table 2:** Apparent Km (Michaelis constant) and Vmax (maximum velocity) and mode of inhibition of α-glucosidase and pancreatic lipase.

α-glucosidase			
	Km	Vmax	Mode of inhibition
Control	8.67 ± 6.58^a^	2.81 ± 1.45^a^	–
EAEP skim (2 mg mL^−1^)	0.17 ± 0.02^b^	0.02 ± 0.004^b^	Uncompetitive
Lipase			

	Km	Vmax	Mode of inhibition
Control	0.24 ± 0.01^a^	0.68 ± 0.01^a^	–
AEP skim (20 mg mL^−1^)	0.17 ± 0.01^b^	0.62 ± 0.02^b^	Uncompetitive
EAEP skim (5 mg mL^−1^)	0.19 ± 0.02^b^	0.59 ± 0.02^b^	Uncompetitive
HEX-AEP skim (20 mg mL^−1^)	0.19 ± 0.01^b^	0.61 ± 0.02^b^	Uncompetitive

Different letters indicate a significant difference (p < 0.05) among the different samples and control for Km or Vmax. Data represent the mean ± standard deviation of triplicates.

For uncompetitive inhibition, the inhibitor binds the enzyme–substrate complex in an allosteric site and has no substrate-like structure. The uncompetitive inhibitor causes a structural distortion of the active and allosteric sites of the complexed enzyme, which prevents the catalysis to occur and results in a decrease in Km and Vmax values^41^. According to Leskovac^42^, uncompetitive inhibition works better in the presence of high concentrations of substrate, a condition likely to occur in the digestive tract during digestion. EAEP skim inhibition of α-glucosidase produced a Km over 50 times lower and a Vmax around 140 times lower than the control. These results indicate that the peptides generated by the proteolysis of almond protein showed a strong ability to bind to the α-glucosidase-substrate complex. Thereby, EAEP skim may be an excellent pool of peptides to delay the breakdown of dietary carbohydrates and consequently reduce the rate of glucose release at the small intestine^15^. Pancreatic lipase inhibition at AEP showed Vmax approximately 10% lower than the control and Km up to 40% lower than the control, showing a weak binding between inhibitors and enzyme–substrate complex.

The uncompetitive inhibition of α-glucosidase by peptides was also observed by Ibrahim et al^15^, who identified two active α-glucosidase inhibitory peptides—SVPA and SEPA. SVPA acted as an uncompetitive inhibitor and SEPA was a non-competitive inhibitor against α-glucosidase. In contrast, Awosika and Aluko^9^ studied yellow field pea protein-derived peptides and found a non-competitive inhibition of α-glucosidase. The uncompetitive inhibition is considered as a rare type of inhibition that may occur in multimeric enzymes^41^. To the best of our knowledge, the first study on inhibition of pancreatic lipase by food-derived bioactive peptides was published by Ngoh and Gan^10^ and dealt with bean protein hydrolysates. Therefore, there is scant information relating peptides and pancreatic lipase inhibition, especially on the enzyme kinetics. Most of the studies regarding lipase kinetics are related to phenolic compounds, such as the study from, Park et al.^40^ who identified a non-competitive or competitive inhibition against lipase at flavonol-3-O-glycosides and flavonol aglycones in *Polygonum aviculare* L., respectively.

Overall, the EAEP skim presented a high inhibitory activity against α-glucosidase and AEP, EAEP, and HEX-AEP skims showed varying degrees of inhibitory activity against lipase. Unlike the α-glucosidase inhibitory effect, AEP skim showed higher inhibitory activity against pancreatic lipase compared with EAEP skim. However, it must be taken into account that the concentration of the EAEP skim (5 mg mL^−1^) was 4 times lower than the active concentration of the AEP skim (20 mg mL^−1^). The lower concentration needed by the EAEP skim can be addressed to its chemical reaction order. EAEP skim presented a zero-order behavior, which means a constant rate of inhibition, independent of the concentration tested. The zero-order kinetics occurs at the limit where the enzyme is saturated with substrate and an increase in the initial substrate concentration will have no effect on the rate of reaction, as no free enzyme is available^42^. The result indicates that the protein fraction extracted from the almond cake by the EAEP has the potential to be used as a source of bioactive protein/peptides to control hyperglycemia and obesity. EAEP skim can retard the release of glucose from complex dietary carbohydrates and could thus be subsequently evaluated regarding potential hyperglycemia reductions. All skim samples may partially suppress and delay the triacylglycerol digestion and consequently help to control hyperlipidemia and obesity^15,36^.

### Antimicrobial activity

The antimicrobial potency of the extracts against five bacteria strains was evaluated by agar disk-diffusion, which measures the formation of a halo, known as the zone of inhibition, and also by broth microdilution, that indicates the minimum inhibitory concentration (MIC). The samples were tested by agar disk-diffusion at a concentration of 20 mg mL^−1^. None of the skim fractions showed inhibition by this method, as they did not cause the formation of inhibition zones (halos). Although the positive control (amoxicillin) did. To eliminate the hypothesis that the concentration tested was too low to exert some antimicrobial activity, all samples were tested against the same bacterial strains at the concentration of 70 mg mL^−1^, by the broth dilution method. Nevertheless, the samples did not exhibit antimicrobial activity against the five strains tested. The theoretical in silico hydrolysis and BIOPEP search did not reveal any antibacterial, antiviral or antifungal peptides, corroborating the in vitro assays.

Different extracts (water, methanol, and ethanol) of bitter almonds were tested against human pathogenic bacteria by Gomaa^29^. The authors observed inhibition activity against only 4 of the 11 microorganisms tested. They attributed the significant inhibitory effect to the phenolic compounds in the extracts. The antimicrobial activity of almond skin^43^, almond oil^44^, and cold press edible oil byproduct^11^ has already been tested. According to the literature, a plant extract depends on which part of the plant was evaluated, the method and solvent used for extraction and finally the concentration tested to be a potent antimicrobial extract^11^.

### In vitro protein digestibility

The profile of each skim protein fraction during oral, gastric, and intestinal digestion was evaluated by SDS-PAGE (Fig. 4). SDS-PAGE showed similar initial protein profiles for AEP and HEX-AEP skims, presenting proteins with a molecular mass between 21.5 and 45 kDa, although some low molecular weight protein bands may be also observed (Fig. 4—Dig 01). On the other hand, for the EAEP skim, a concentration of low molecular mass proteins and peptides at 14.4 kDa and below became evident. Dig 02 (Fig. 4) shows the profile for samples that received salivary solution (SSF) plus gastric solution (SGF), immediately after addition, Dig 03 shows SSF plus SGF after 2 h. Overall, there was little difference in the same sample, between Dig 02 and Dig 03, indicating fast digestion of the extracted almond cake proteins by pepsin. In a previous study regarding almond protein digestibility^45^, pepsin was able to hydrolyze the proteins in 5 min, indicating rapid hydrolysis capability. Although EAEP showed initially an already digested profile due to the partial hydrolysis caused by the use of protease during the extraction, the addition of pepsin seemed to reduce the peptide cluster at the bottom of the gel—below 14.4 kDa, indicating further digestion and formation of lower mass peptides. Dig 04 (Fig. 4) presents the profile of the samples containing salivary solution (SSF), gastric solution (SGF) and also the intestinal solution (SIF), after another 2 h reaction. At this stage, all samples showed similar profiles, with the higher molecular mass bands probably derived from the different proteins in the pancreatin solution added. The results indicate that both pepsin and pancreatic proteases were efficient in hydrolyzing almond cake proteins independent of the extraction method.

Simulated digestion includes an oral, a gastric and an intestinal phase. These methods involve the use of digestive enzymes and their concentrations as well as the pH, digestion time, and salt concentrations to simulate physiological conditions *in vivo*^46^. After each digestion phase, samples must be analyzed to quantify the amount of the initial protein that was hydrolyzed by the digestive proteases added. There are several methods for chemically determining the hydrolysis of proteins such as pH–stat, o-phthaldialdehyde (OPA) and trichloroacetic acid soluble nitrogen (SN-TCA) methods. The pH–stat method is based on the amount of protons released during hydrolysis; the OPA method is based on the measurement of amino groups generated by the hydrolysis and the SN-TCA method measures the amount of TCA-soluble nitrogen released by the hydrolysis^47^. The pH–stat and OPA methods are considered quick and simple methods for quantifying the degree of hydrolysis (DH) of protein hydrolysates and allow for real-time monitoring of the hydrolysis reaction as it proceeds. However, the accuracy of DH values obtained by the pH–stat and OPA methods depends on the type of enzyme activity and on the protein substrate, respectively. The pH–stat method may underestimate DH if the enzyme shows high exopeptidase activity. In the OPA method, unstable products are formed by reacting with cysteine and proline residues and this method also requires a derivatization step^47,48^. The SN-TCA method does not determine the number of peptide bonds cleaved, so this method does not determine the DH directly, it measures the TCA-soluble nitrogen generated by the hydrolysis, which is supposed to consist only of amino acids and small peptides. The SN-TCA method is simple and rapid to perform, however, the assumption that TCA-soluble nitrogen is composed only by small peptides and amino acids may not be correct, since not all intact proteins are precipitated by TCA^53^. Considered a suitable method by several authors^53,49−51^, the SN-TCA method was applied in this study to compare the effect of either EAEP or AEP on the digestibility of the protein (skim fraction) extracted.

Among the tested samples, the highest digestibility was found for the HEX-AEP (85%) skim, followed by the AEP skim (73%) and the EAEP skim (64%). The high digestibility regardless of the extraction is consistent with a previous True Protein Digestibility (% TPD) in vivo test from Ahrens et al.^52^, who found digestibility from 82 to 92% depending on the varieties of the whole almonds. Moreover, Sathe^53^ and Sze-Tao and Sathe^45^ also reported high in vitro almond protein digestibility. The high digestibility of HEX-AEP skim may be explained by the removal of the residual oil by hexane extraction (in a Soxhlet). During the hexane extraction, the almond cake was subjected to mild temperatures (68–70 °C) which might have resulted in protein denaturation. In some situations, some degree of protein denaturation might improve enzyme accessibility to the protein thus improving the digestibility^54^. Conversely, the EAEP skim presented the lowest digestibility despite its higher initial DH. Typically, digestibility increases with protein hydrolysis^55^. However, the extensive hydrolysis achieved by the EAEP (DH = 23%) entailed fewer attack sites available to the digestion enzymes (pepsin and pancreatin), which led to an underestimation of this parameter^55^. Similar results were found by Betancur-Ancona et al.^55^, who reported higher digestibility in the raw materials (*Phaseolus lunatus*) than for the hydrolyzates produced.

## Conclusion

The effects of the aqueous (AEP) and enzyme-assisted aqueous extraction processes (EAEP) on the biological properties of the AEP and EAEP skims were evaluated. The use of an enzyme to assist the extraction of the almond cake resulted in the production of a skim fraction with higher TPC and the presence of bioactive peptides associated with increased antioxidant activity and inhibitory effects against α-glucosidases. The in silico theoretical hydrolysis indicated the presence of 4 antioxidant peptides, 13 peptides with structural requirements for α-glucosidase inhibition and 16 highly hydrophobic peptides, likely to inhibit pancreatic lipase. The AEP skim presented a similar profile as the HEX-AEP skim regarding their antioxidant capacity, TPC and digestibility, but provided the highest lipase inhibition potential, and therefore could be used in the prevention of obesity. Although further studies are required to characterize the active compounds and the mechanisms of action associated with the observed bioactivities, the present study demonstrated that the almond cake can be transformed into value-added health-promoting products by the application of environmentally friendly extraction processes.

## Supplementary information

Supplementary file1

Supplementary file2
