# Supplementary material for: Biological properties of almond proteins produced by aqueous and enzyme-assisted aqueous extraction processes from almond cake
Source: Sci Rep. 2020 Jul 2;10:10873. doi: 10.1038/s41598-020-67682-3 (PMC7331752; doi:10.1038/s41598-020-67682-3)
Supplement: Supplementary file 1 — Supplementary file1 [file 41598_2020_67682_MOESM1_ESM.pdf]

Supplementary Information for:

Biological properties of almond proteins produced by aqueous and enzyme-assisted aqueous extraction processes from almond cake

Thaiza S. P. de Souza[1,2], Fernanda F. G. Dias[1], Joana Paula S. Oliveira[2], Juliana M. L. N. de Moura Bell[1,3], Maria Gabriela B. Koblitz[2]

[1] Department of Food Science and Technology, University of California, Davis, One Shields Avenue, Davis, CA, 95616, United States

[2] Food and Nutrition Graduate Program, Federal University of the State of Rio de Janeiro, Rio de Janeiro, RJ, 22290-240, Brazil

[3] Biological and Agricultural Engineering, University of California, Davis, One Shields Avenue, Davis, CA, 95616, United States

TABLE S1: Summary of resultant antioxidant peptides from the major almond protein subunits after subtilisin digestion *in silico* using BIOPEP database

Amandin subunit	ID	Sequence	Location	Name	Function	Activity	MI Mass	Chemical mass
Prunin-1	3317	HL	[81-82]	*	Antioxidative	Antioxidative	268.1420	268.3020
	7873	IY	[92-93]	peptide from soybean protein isolates: beta-conglycinin and glycinin	Antioxidative	Antioxidative	294.1470	294.3300
	8037	PWH	[429-431]	synthetic peptide	*	Antioxidative	438.1890	438.4720
	8224	VY	[439-440]	antioxidative peptide	Free radical scavenging	Antioxidative	280.1310	280.3030
Prunin-2	3317	HL	[72-73]	*	Antioxidative	Antioxidative	268.1420	268.3020
	7873	IY	[83-84]	peptide from soybean protein isolates: beta-conglycinin and glycinin	Antioxidative	Antioxidative	294.1470	294.3300
	8224	VY	[386-387]	antioxidative peptide	Free radical scavenging	Antioxidative	280.1310	280.3030

H – histidine; L – leucin; I – isoleucine; Y – tyrosine; P – proline; W – tryptophan; V – valine. Digestion simulation was conducted with subtilisin in the BIOPEP database [1]

TABLE S2: Overview of the presence of specific amino-acids suggested as important requirement for the α-glucosidase inhibition activity

Amandin subunit	Sequencie	R, L, S, T or Y at N-terminus	A or M at C-terminus	P close to C-terminus
Prunin-1	QQRR**A**	no match	**A**	no match
	**R**AL	**R**	no match	no match
	**R**NGIY	**R**	no match	no match
	**R**QL	**R**	no match	no match
Prunin-2	IPQNH**A**	no match	**A**	no match
	**R**ADF	**R**	no match	no match
	**R**AL	**R**	no match	no match
	**R**IS	**R**	no match	no match
	**R**PS	**R**	no match	no match
	**R**QHIF	**T**	no match	no match
	**T**NTL	**T**	no match	no match
	**T**PHW	**T**	no match	no match
	N**P**S	no match	no match	**P**
	R**P**S	no match	no match	**P**

A – alanine; M – methionine; P – proline; R – arginine; K – lysine; S – serine; T – threonine; Y – tyrosine. The criteria used was suggested by Ibrahim *et al.* [2], that proposed the structural requirements for α-glucosidase inhibition as tri – to hexapeptides with serine S, T, Y, L or A as the ultimate N-terminal residue and P preferably at the penultimate C- terminal position while A or M at ultimate C-terminal position.

TABLE S3: Overview of the presence of specific amino-acids suggested as important requirement for the lipase inhibition activity

Amandin subunit	Sequence	Hydrophobicity (%)	Hydrophobic residues
Prunine-1	V**AIPAG**	83.33	A, P, G
	M**A**K**AF**	80.00	A, F
	**A**Q**AL**	75.00	A, L
	R**AL**	66.67	A, L
	**MA**N**GL**	60.00	M, A, G, L
	N**AP**Q**L**	60.00	A, P, L
	**GA**	50.00	G, A
Prunine-2	**L**	100.00	L
	**W**	100.00	W
	**A**Q**AL**	75.00	A, L
	**A**R**L**	66.67	A, L
	P**AG**	66.67	A, G
	R**AL**	66.67	A, L
	VN**AP**Q**L**	66.67	A, P, L
	**AG**	50.00	A, G
	**GA**	50.00	A, G
	QN**AF**	50.00	A, F
	R**A**D**F**	50.00	A, F

A – alanine; L – leucine; P – proline; F – phenylalanine; G – glycine; M – methionine; W – tryptophan. The criteria used was suggested by Ngoh et al. [3], that proposed the presence of hydrophobic residues as characteristic of a lipase inhibitor. To evaluate the hydrophobicity of the sequences, the platform: Peptide Hydrophobicity/Hydrophilicity Analysis Tool, was used.

Supplementary References:

1. Minkiewicz, P., Iwaniak, A. & Darewicz, M. BIOPEP-UWM Database of Bioactive Peptides: Current Opportunities. *Int. J. Mol. Sci.* **20**, 2–23 (2019).
2. Ibrahim, M. A., Bester, M. J., Neitz, A. W. H. & Gaspar, A. R. M. Structural properties of bioactive peptides with α-glucosidase inhibitory activity. *Chem Biol Drug Des* **91**, 370–379 (2018).
3. Ngoh, Y.-Y. & Gan, C.-Y. Enzyme-assisted extraction and identification of antioxidative and α-amylase inhibitory peptides from Pinto beans (Phaseolus vulgaris cv. Pinto). *Food Chem* **190**, 331–337 (2016).
