# Supplementary figures and images for: Biological properties of almond proteins produced by aqueous and enzyme-assisted aqueous extraction processes from almond cake
Source: Sci Rep. 2020 Jul 2;10:10873. doi: 10.1038/s41598-020-67682-3 (PMC7331752; doi:10.1038/s41598-020-67682-3)

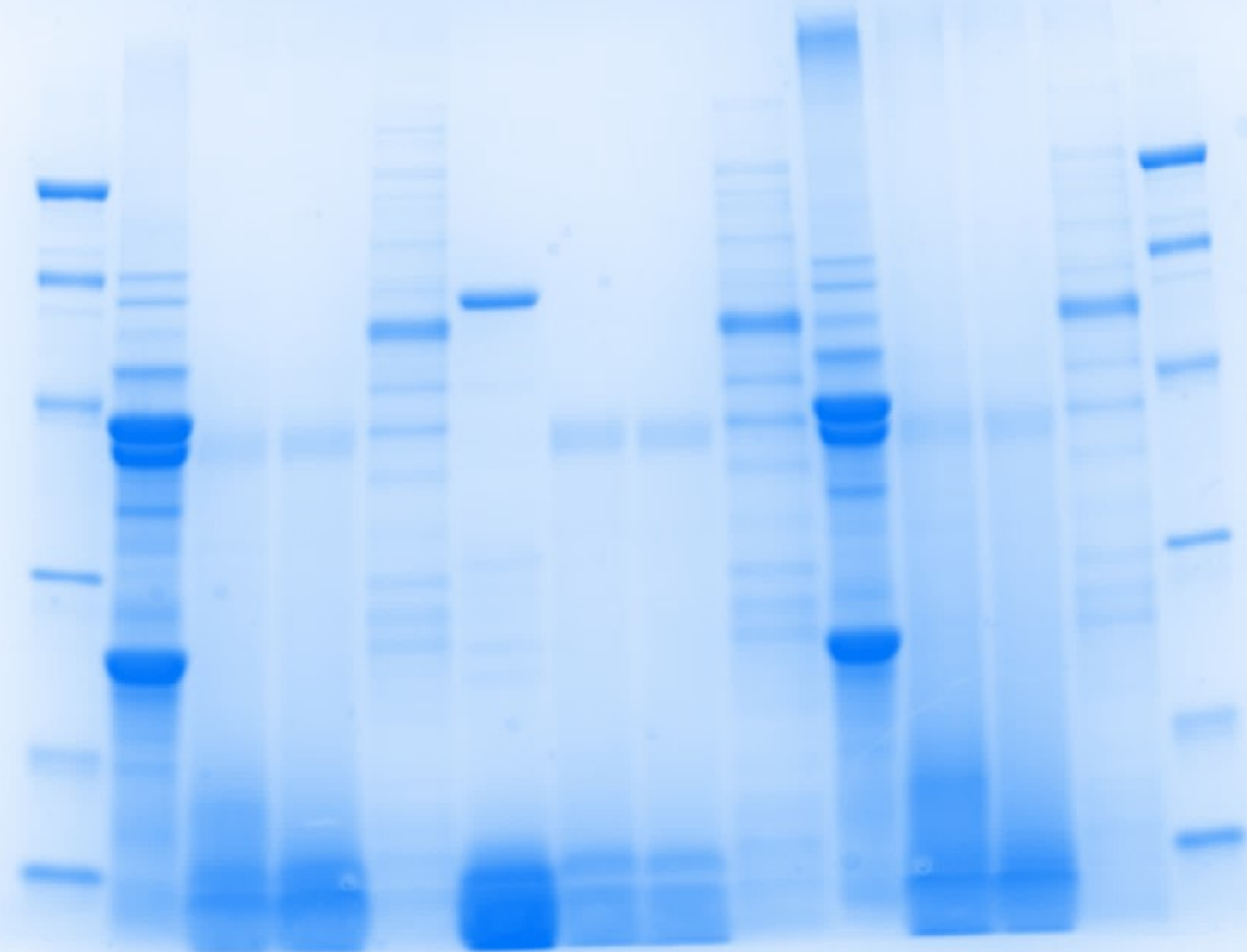

Supplement: Supplementary file 2 — Supplementary file2 [file 41598_2020_67682_MOESM2_ESM.pdf]
